# Assessing Drivers of Full Adoption of Test and Treat policy for Malaria in Senegal

**DOI:** 10.4269/ajtmh.14-0595

**Published:** 2015-07-08

**Authors:** Christina Faust, Jonathan Zelner, Philippe Brasseur, Michel Vaillant, Malick Badiane, Moustafa Cisse, Bryan Grenfell, Piero Olliaro

**Affiliations:** Department of Ecology and Evolutionary Biology, Princeton University, Princeton, New Jersey; Fogarty International Center, National Institutes of Health, Bethesda, Maryland; UMR 198 Institut de Recherche pour le Développement (IRD), Senegal; Methodology and statistics Unit (CCMS) Centre de Recherche Public (CRP)–Santé, Strassen, Luxembourg; District Médical d'Oussouye, Oussouye, Sénégal; Programme National de Lutte contre le Paludisme (PNLP), Ministère de la Santé et de la Prévention, Rue Aimé Césaire, Dakar, Sénégal; Woodrow Wilson School, Princeton University, Princeton, New Jersey; UNICEF/UNDP/WB/WHO Special Programme for Research and Training in Tropical Diseases (TDR), Switzerland; Centre for Tropical Medicine, University of Oxford, Oxford, United Kingdom

## Abstract

Malaria treatment policy has changed from presumptive treatment to targeted “test and treat” (T&T) with malaria rapid diagnostic tests (RDTs) and artemisinin combination therapy (ACT). This transition involves changing behavior among health providers, meaning delays between introduction and full implementation are recorded in almost every instance. We investigated factors affecting successful transition, and suggest approaches for accelerating uptake of T&T. Records from 2000 to 2011 from health clinics in Senegal where malaria is mesoendemic were examined (96,166 cases). The study period encompassed the implementation of national T&T policy in 2006. Analysis showed that adherence to test results is the first indicator of T&T adoption and is dependent on accumulation of experience with positive RDTs (odds ratio [OR]: 0.55 [*P* ≤ 0.001], 95% confidence interval [CI]: 0.53–0.58). Reliance on tests for malaria diagnosis (rather than presumptive diagnosis) followed after test adherence is achieved, and was also associated with increased experience with positive RDTs (OR: 0.60 [*P* ≤ 0.001], 95% CI: 0.58–0.62). Logistic models suggest that full adoption of T&T clinical practices can occur within 2 years, that monitoring these behavioral responses rather than RDT or ACT consumption will improve evaluation of T&T uptake, and that accelerating T&T uptake by focusing training on adherence to test results will reduce overdiagnosis and associated health and economic costs in mesoendemic regions.

## Introduction

Global malaria treatment policy has changed from a system of widespread presumptive treatment based on clinical diagnosis of fever to targeted treatment following positive parasitological diagnosis,^1^ also known as “test and treat” (T&T). The long-standing practice of presumptive treatment is being overturned by 1) failure of cheap drugs (i.e., chloroquine, sulfadoxine/pyrimethamine) and their subsequent replacement with more effective, yet expensive, artemisinin combination therapies (ACTs), 2) mounting evidence that malaria only causes a proportion of all fevers in malaria endemic regions,^2^,^3^ and 3) improvements in diagnostic capabilities. Parasitological confirmation followed by ACT has the potential to reduce misdiagnosis, improve patient outcomes, and minimize costs. But this is possible only if T&T policies are implemented efficiently.

Although 41 out of 45 countries in Africa with malaria transmission have adopted World Health Organization (WHO) T&T policies for malaria,^4^ in practice policy is not consistently followed. There are many reports of antimalarial treatments being routinely administered to patients who tested negative on microscopy^5^–^7^ or rapid diagnostic test (RDT).^8^–^11^ In addition, there are many instances where parasitological tests are available but presumptive diagnosis is still often used for prescription of antimalarials.^12^–^14^

T&T policies can now, theoretically, be implemented anywhere because RDTs have significantly expanded testing capabilities. These tests can help reduce overtreatment of malaria and associated costs,^15^ but adherence to test results must improve if T&T strategies are cost-effective.^16^ This potential of RDT programs to reduce unnecessary treatment has been demonstrated by the reduction in ACT consumption following introduction of RDTs in some settings.^13^,^17^–^20^ Although RDTs may have no impact on ACT consumption in areas where microscopy is available,^21^ their use has been shown to reduce unnecessary malaria treatments in areas that previously relied solely on clinical diagnosis,^12^ but complete compliance of T&T has yet to be demonstrated.

In Senegal, access to RDTs has improved diagnostic capabilities nationwide and reduced the amount of antimalarials administered.^18^ Economic analysis suggested that adoption of T&T policies could significantly reduce costs associated with malaria in the region—if adherence to test results was consistent.^22^ Despite these positive reports, delays between the introduction of T&T policies and consistent usage and trust in the results of RDTs demonstrate that there is a need to examine if febrile patients may be managed more effectively.

Our objective was to identify factors influencing the time course of behavioral changes following new T&T policies in mesoendemic regions of Senegal. Specifically, we quantified the delay between the introduction of policies and uptake in six community health centers, as well as the process of conversion to RDT-based treatment. Characterizing the length of this delay and the factors that affect the rate of change in testing behavior is important for evaluating whether T&T policies have been effectively “rolled out” or require additional training and support to be effective. Understanding the sequence of changes that precedes full adoption of T&T policies will help to highlight behaviors that are important for monitoring in areas where implementation has been less successful, and suggest areas of focus to speed uptake of new policies.

## Materials and Methods

### Study sites.

Complete, detailed, and anonymous clinical data on consultations, fevers, malaria tests and results, and treatments were available from five dispensaries in the District of Oussouye, in the southern Casamance region of southwest Senegal, and one dispensary in Toucar (District of Niakhar, in western Senegal) (Table 1). Each dispensary is run by a nurse who is supported by a technician and health workers. The outpatient clinic of Oussouye is housed in the local hospital. A district medical doctor is based in each clinic in Oussouye and Niakhar.

Malaria in these areas is mesoendemic, with an increase of cases during the rainy season (July–December). Interventions to reduce malaria burden have included insecticide-treated nets, indoor residual spraying, and chemotherapies. In the District of Oussouye, malaria risk has dramatically dropped over a 15-year period, and there is no longer a clear age prevalence signature.^23^

**Figure 1.**
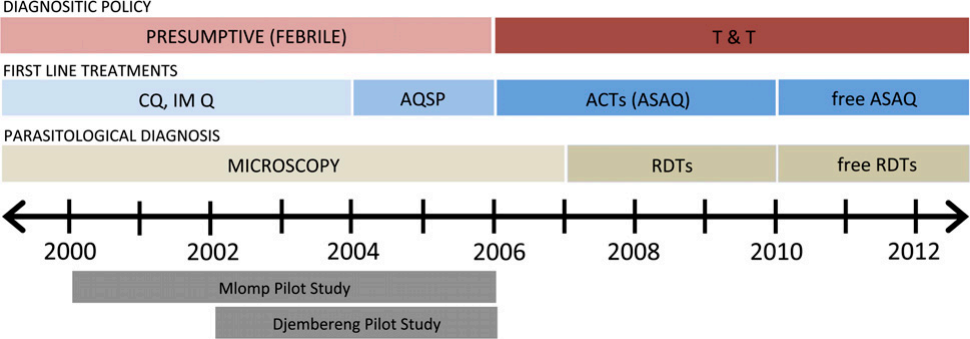
Timeline of malaria policies in Senegal. This timeline illustrates the changes in malaria policies over the period of 2000–2012. Prior to a “test and treat (T&T)” national policy in 2006, malaria was diagnosed by clinical symptoms (usually fever). Over the same time frame the recommended first-line treatment changed from 1) chloroquine (CQ) or intramuscular quinine (IM Q) to 2) amodiaquine (AQ) plus sulfadoxine/pyrimethamine (SP), and finally 3) artesunate plus amodiaquine (ASAQ), a type of artemisinin combination therapy (ACT). Parasitological diagnosis was made by microscopy on Giemsa-stained thick and thin smear until 2007, when rapid diagnostic tests (RDTs) were made available at all sites. Both RDTs and ACTs were made freely available starting in 2010. The two pilot studies are also shown in grey.

A series of policy changes in the diagnosis and treatment of malaria cases took place in this region (Figure 1). In Senegal, the national T&T policy was enacted in 2006. In Oussouye district, two pilot phases preceded national policy in Mlomp (2000–2005)^22^,^24^,^25^ and Djembereng (2002–2005), during which diagnostics (microscopy on thick, counting 200 WBCs, and thin blood smears), treatment (artesunate plus amodiaquine [ASAQ]), and training were provided without cost. All malaria treatments were administered under the supervision of a nurse, and the nurses were trained for 3 days prior to the start of pilot phase to demonstrate protocols for ASAQ treatment administration.

### Measuring T&T policy uptake.

**Figure 2.**
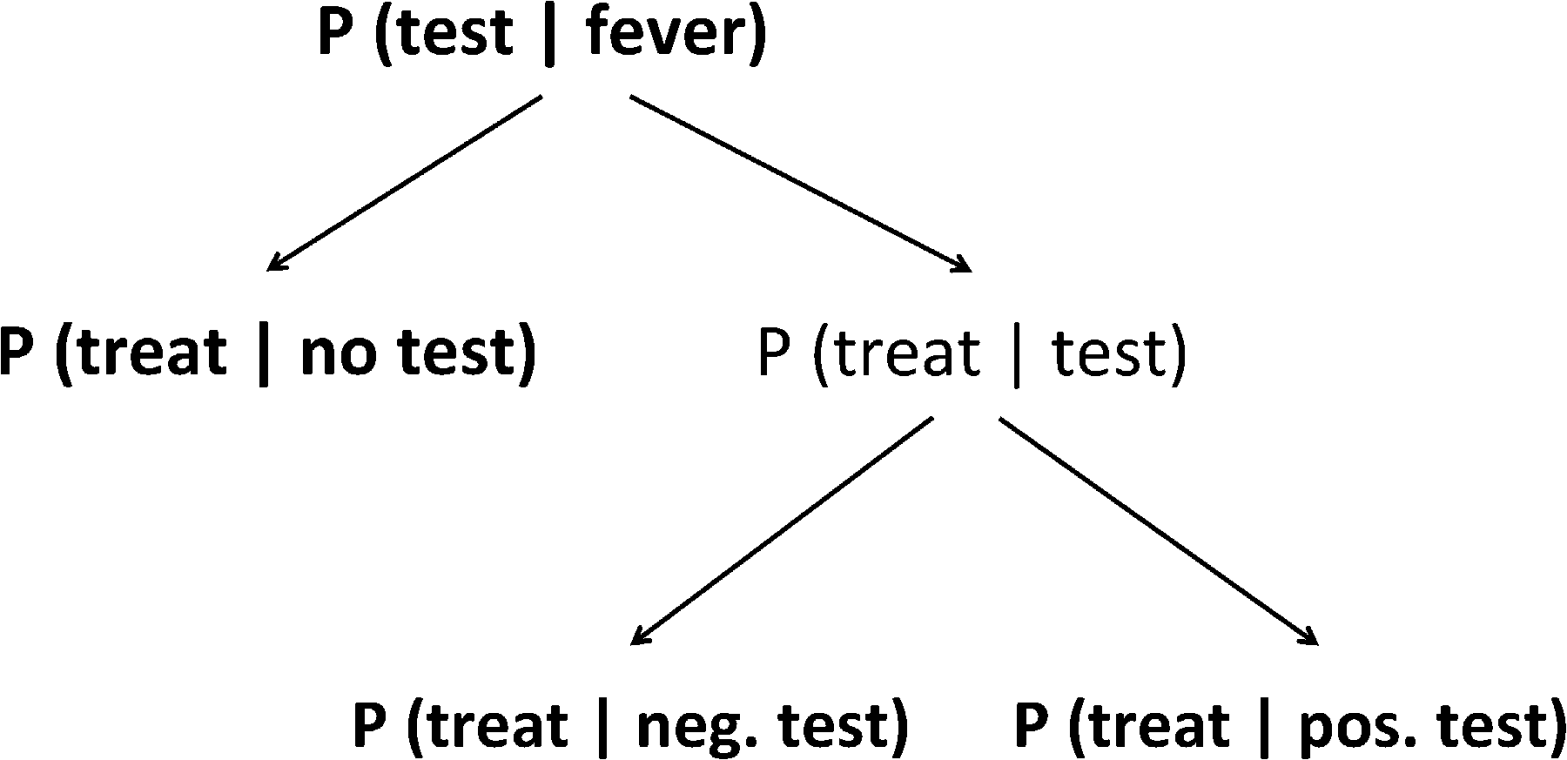
Decision tree for application of test and treat (T&T) policies. Entries in the tree reflect conditional relationships between provider behaviors. For example, the decision to administer antimalarials to an individual with a positive test (pos. test) is conditional on the probability of administering a malaria test. Bolded entries in the tree indicate provider decisions included in our analysis. “Treat” refers to only treatment with antimalarials and not any other chemotherapy (i.e., antibiotics).

The goals of T&T policies are to reduce overtreatment of malaria, encourage correct diagnosis, and reduce costs associated with misdiagnosis. We disentangled this decision process into several behaviors that can be objectively measured from clinical records and conceptualized as a decision tree (Figure 2). At the root of this tree is the initial decision of a health provider to test a patient presenting with fever for the presence of malaria parasites, *P*(*test*∣*fever*); (it literally translates to the probability of testing given a patient with a fever). Conditional on the decision whether to use a test for a febrile patient, the provider then has to make the decision to treat. We denoted the probability of prescribing antimalarial treatment among individuals not administered a test as *P*(*treat*∣*no test*)—when this is equal to 1 prescribers are exclusively using presumptive diagnosis to treat febrile patients, whereas when this is equal to 0 prescribers are only administering treatment to tested individuals. Among those who were tested for malaria, we denoted the probability of treatment of individuals with a positive test as *P*(*treat*∣*pos. test*), with a negative test as *P*(*treat*∣*neg. test*). We defined optimal adherence to T&T as *P*(*treat*∣*no test*) = 0, *P*(*treat*∣*pos. test*) = 1, and *P*(*treat*∣*neg. test*) = 0. In words, the objectives of malaria T&T policy are to test all suspected cases of malaria, treat all positive test results, and not treat negative malaria cases with antimalarials. These probabilities can be used for evaluation in any setting to determine effectiveness of policies.

To understand treatment decisions in detail, we also analyzed the types of antimalarial treatments prescribed to patients. ASAQ (a type of ACT) has been the recommended first-line treatment since 2006, but other antimalarials were available at dispensaries (including chloroquine, quinine, and AQ/sulfadoxine/pyrimethamine [SP]), despite resistance. We examined the probability of ACT administration compared with other antimalarials prescribed, *P*(*ACT*∣*antimalarials*). These treatments included individuals that received antimalarials regardless of testing or outcome of the test.

### Statistical analysis.

We first tested these data to see whether behavioral responses were well described by a logistic model (Supplemental Equation 1). The logistic model is useful for describing the rate of change from one regimen to another, for example, the change from presumptive diagnosis to a full T&T regime. Parameters describing the rate of change and the midpoint of the transition were estimated for each dispensary and response via Markov chain Monte Carlo (MCMC) using PyMC 2.3^26^ in Python 2.7.4. Data for two clinics were available for every month from 2000 to 2011 (Mlomp and Djembereng), while the remaining clinics cover subsets of this period (Table 1).

To evaluate factors that affected the timing and rate of transition, we fitted logistic regression models to the data using the generalized linear model function (*glm*) in R version 3.0.2 (Vienna, Austria).^27^ All districts were analyzed together, the outcome responses are behaviors bolded in Figure 2 and *P*(*ACT*∣*antimalarials*). Models were chosen using Akaike information criterion (AIC).^28^ Adjusted odds ratios (ORs) were calculated for the best-fit models.

Predictor variables and model selection are described in detail in the supplementary material, but we will briefly mention important predictors here. Data from each month were available—so predictor variables reflect cumulative monthly values. Data that showed significant seasonality were normalized as anomalies (Supplemental Figures 1 and 2)—for example, rainfall anomalies were calculated by finding the mean rainfall and monthly anomalies represented standard deviations from this mean. Because patient load, treatment seeking behavior, and timing of RDT availability varied significantly between district health centers, we used relative accumulation of tests as a proxy for experience with RDTs and/or microscopy. For example, accumulation of positive tests (RDTs plus microscopy tests) was calculated for each district by summing the number of positive tests over the entire period, then dividing the cumulative positive tests each month by that sum of all positive tests. Values were then multiplied by 10, so that each unit increase represented a 10% accumulation of positive tests and gave a more interpretable metric (Supplemental Table 1 and Supplemental Figure 3).

### Ethics.

The pilot intervention study was approved by the Ethic Committee of Institut Pasteur of Dakar. The collection of anonymous administrative data was conducted under the authority of the district medical officer, as required locally. The funding sources of the study had no role in study design, data collection, data analysis, data interpretation, or writing of the report.

## Results

### Transitions to T&T.

**Figure 3.**
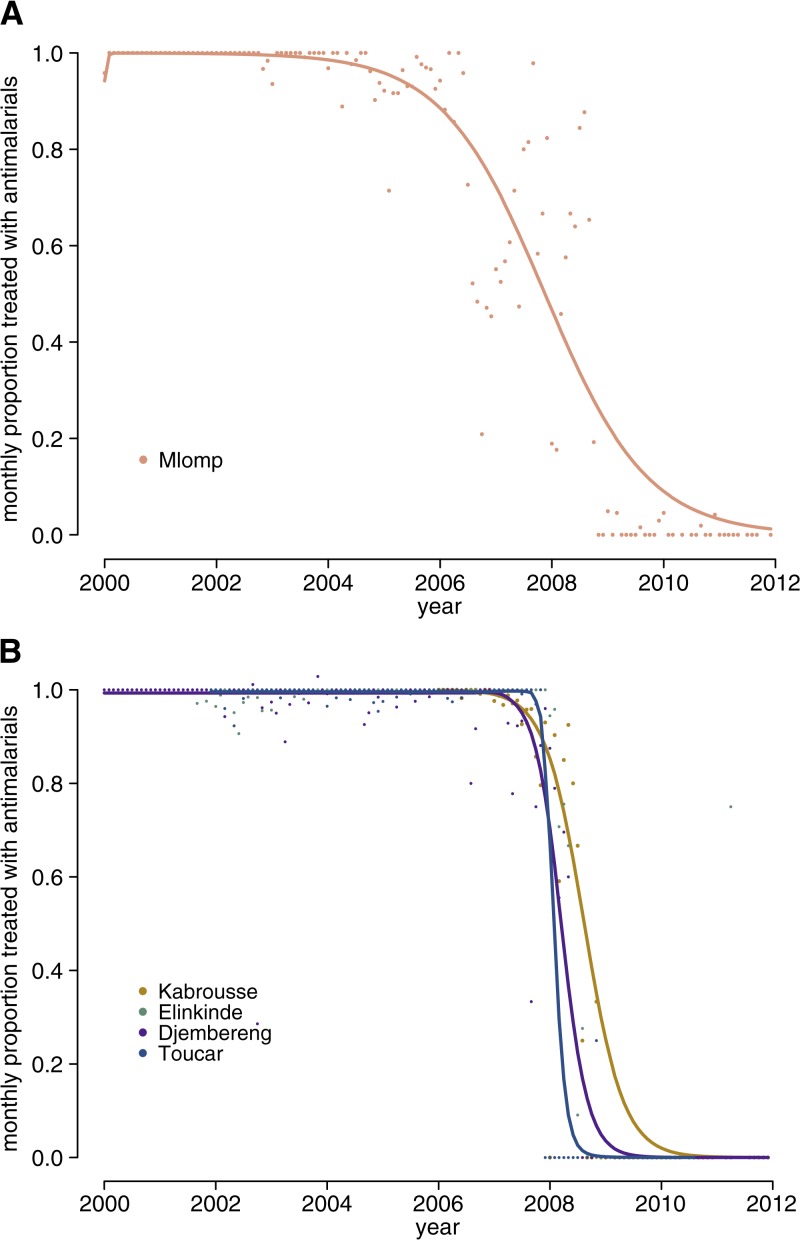
Transitions in clinical behavior. (**A**) Adherence to rapid diagnosis test (RDT) results in Mlomp, Senegal. The points on the graph represent the proportion of cases with negative RDTs, who received antimalarials. The proportion begins to drop during the pilot phase, but complete adherence is not obtained until 2009. The trend is well represented by a logistic model. (**B**) Proportion of clinically diagnosed cases receiving antimalarials: non-pilot clinics. The proportion of untested febrile cases that are treated with antimalarials drops significantly in all four districts and the rapid change in behavior can be represented with a logistic model.

The logistic model was appropriate to describe the transition of some clinical behaviors, but not for all outcomes or districts. It accurately described the transition from zero to full adherence of test results (*P*(*treat*∣*neg. test*) = 1) in Mlomp, where a pilot study took place (Figure 3A

); this transition began before national policies and took almost 4 years for saturation (complete and sustained adherence to test results). In other districts, there was no clear transition from a regime in which treatment decisions were not dependent on test results to one where they consistently followed test results. However, accumulation of positive RDT results was a strong predictor of behavior change (Table 2 ; OR: 0.55 [*P* < 0.001], 95% confidence interval [CI]: 0.53–0.58), which positively correlated with time (see Supplemental Figure 2) and indicated that a similar mechanism may account for these changes in adherence across districts.

**Figure 4.**
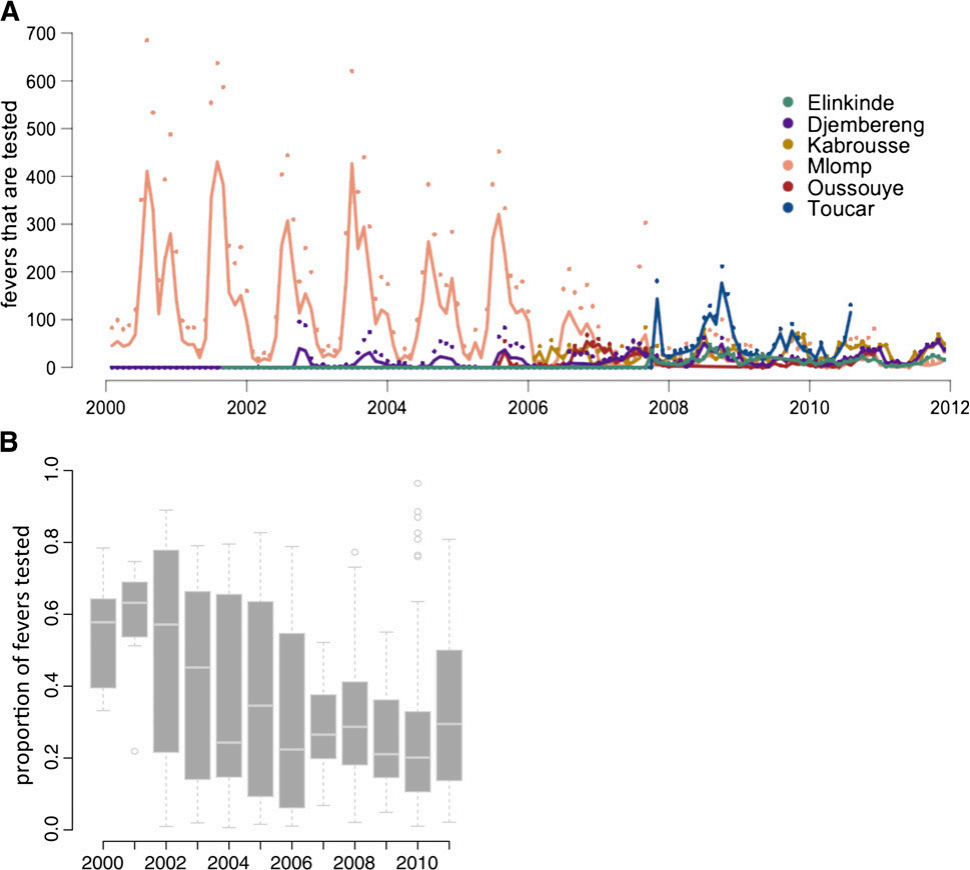
Testing of febrile cases. (**A**) The best-fit logistic regression model for *P*(*test*∣*fever*) is shown in for all of the districts. The line is the model, whereas the points are the data. (**B**) Although testing rates are initially high, the probability of testing a febrile case decreases over time, likely reflecting the realization that only a portion of fever cases are caused by malaria.

The transition to consistent reliance on testing (*P(treat*∣*no test)*) was well described by the logistic regression in Djembereng, Elinkinde, Kabrousse, and Toucar (Figure 3B). The transition period lasted between 12 and 24 months. Change in the probability of testing fevers was not well described by the logistic regression; testing rates fluctuated over time and were determined by many factors (Figure 4 and Table 2).

### Clinical behavior in detail.

Testing behavior, *P*(*test*∣*fever*), represents the initial decision made by a health provider when presented with a febrile patient. To comply with T&T guidelines, health providers should test patients before they treat with antimalarials, but because only a portion of fevers are malaria cases, testing all febrile cases is not necessary for T&T compliance. National T&T policies (OR: 16.72 [*P* < 0.001]; 95% CI: 13.39–21.18), pilot phases (OR: 73.16 [*P* < 0.001]; 95% CI: 58.66–92.64), availability of RDTs (OR: 5.60 [*P* < 0.001], 95% CI: 5.05–6.22), and monthly rainfall (OR: 1.13 [*P* < 0.001]; 95% CI: 1.11–1.14, per one standard deviation increase of rain [∼17 cm]) all increased the probability of testing febrile cases (Table 2). Accumulating experience with positive tests (both RDTs and microscopy) increased testing (OR: 1.17 [*P* < 0.001], 95% CI: 1.16–1.18), but this effect was diminished when there was access to RDTs (Figure 4B). The model suggested that health practitioners are relying on seasonal environmental cues (rainfall) to supplement national and local policies when deciding to test patients. Although testing initially increased, it eventually decreased over time, potentially influenced by the declining number and proportion of positive malaria cases (Supplemental Figure 4).

**Figure 5.**
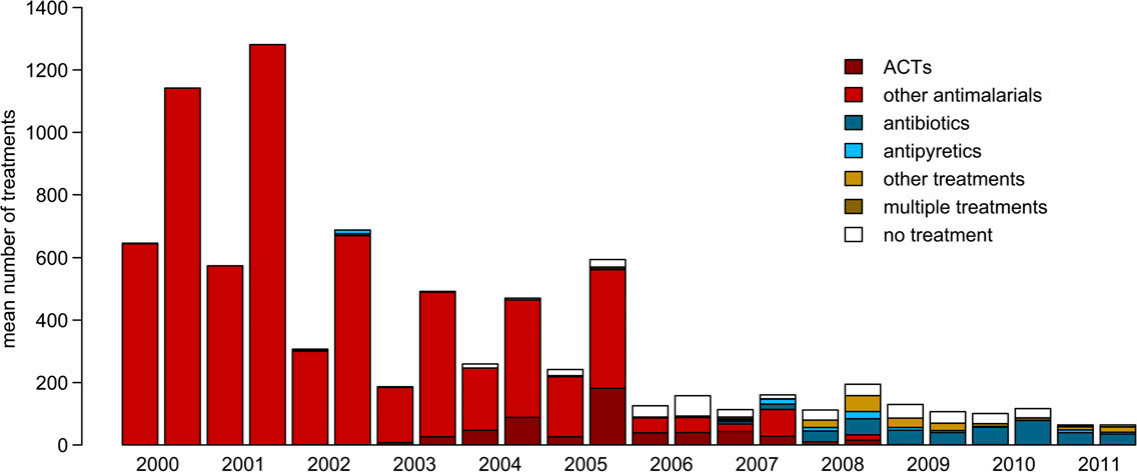
Treatment of negative fever cases. The treatment of negative fever cases changes drastically over time. We show proportion treatments in each class that are given to negative malaria patients. To simplify the figure, data are shown by season rather than monthly. Until national test and treat (T&T) policies in 2006, almost all negative malaria treatments received some kind of antimalarial. A year after the national T&T policy, antibiotics begin to be administered to fevers negative for malaria and the proportion increases as time progresses. In addition, fevers were rarely released without some form of treatment.

In all districts, cases with a positive test result (either microscopy or RDTs) were always treated with antimalarials. In addition, cases that tested negative for malaria were rarely released without some intervention, either antimalarials, antibiotics, antipyretics or other treatment (Figure 5). The probability that health providers will still treat negative test results with antimalarials increased with febrile cases in that month (OR: 1.90 [*P* < 0.05], 95% CI: 1.75–2.07) and during pilot phases (OR: 28.00 [*P* < 0.001], 95% CI: 22.45–35.09) (Table 2). This means that clinicians were assimilating local information, such as pilot phases or case load, and used it to overrule test results. However, accumulation of provider experience with reliable measurements (accumulation of positive RDTs was used as a proxy) decreased the probability of administering antimalarials to negative individuals (OR: 0.55 [*P* < 0.001], 95% CI: 0.53–0.58). It is tempting to conclude that increased experience with positive RDTs reinforced health practitioners trust in the results, and lead to adherence to test results. Accumulation of test results was not linear (see Supplemental Figure 3) and was the best predictor in multivariate models, suggesting that the experience with positive tests rather than just time is important for promoting adherence. Because microscopy diagnostics were prevalent during the pilot phases, it is difficult to disentangle if the usage of microscopy impacted the probability of treatment of negative tests compared with RDTs.

The last behavioral transition is the abandonment of presumptive diagnosis for malaria cases. In all of the dispensaries, complete dependence on parasitological tests, *P*(*treat*∣*no test*) = 0), followed full adherence to test results, *P*(*treat*∣*neg. test* = 0). Monthly fevers greater than average increased the likelihood that an untested individual (OR: 1.19 [*P* < 0.001], 95% CI: 1.16–1.22) or one with a negative test (OR: 1.90 [*P* < 0.05], 95% CI: 1.75–2.07) received treatment. Health practitioners were using local febrile patient loads to help access the risk of malaria—this made sense when malaria was major causative agent for malaria, and reflects the history of presumptive diagnosis. However, availability of RDTs (OR: 0.47 [*P* < 0.001], 95% CI: 0.41–0.54) and increased experience with positive RDTs significantly decreased reliance on presumptive diagnosis (OR: 0.60 [*P* < 0.001], 95% CI: 0.58–0.62). Again, pilot phases negatively impacted behaviors and actually increased the probability of treating untested individuals (OR: 1.30 [*P* < 0.001], 95% CI: 1.15–1.46).

### Choice of antimalarials.

**Figure 6.**
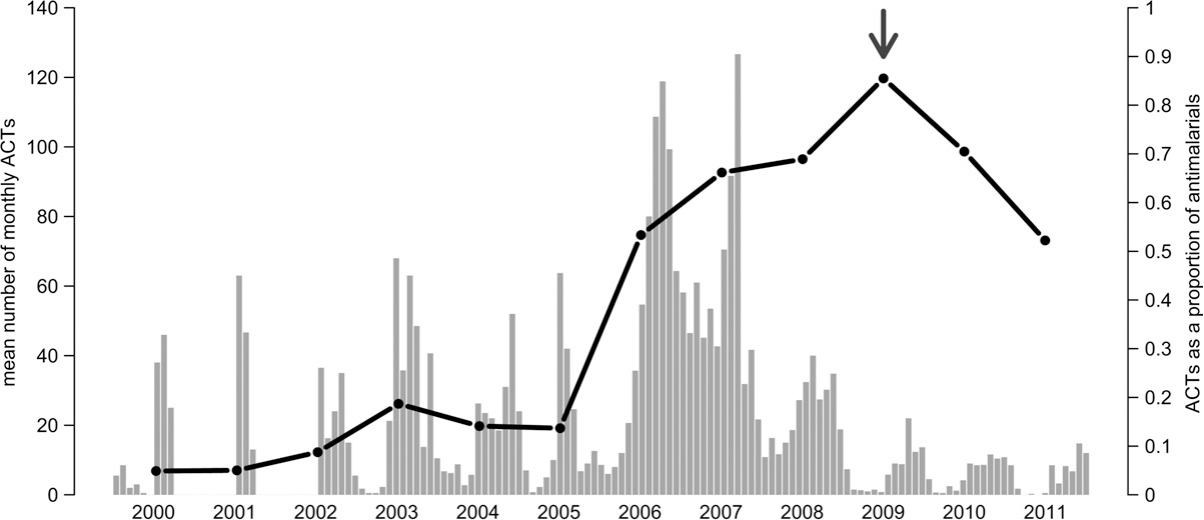
Artemisinin combination therapy (ACT) usage. The total number of ACTs increases (gray bars) immediately after the national test and treat (T&T) policy is enacted, as does the proportion of antimalarials that are ACTs (black line). However, the proportion of ACTs administered does not equal 1.0 during the study period, even when all clinics are following T&T policies (indicated by gray arrow), in fact, proportion of ACTs decreases after clinical behaviors adhere to T&T guidelines.

The frequencies of ACT administration compared with other antimalarials prescribed, *P*(*ACT*∣*malaria treatment*), varied over time and between clinics. The overall trend was fewer total antimalarial treatments over time and increased proportion of ACTs that were administered; however, none of the districts prescribed 100% antimalarials as ACTs (Figure 6

). Monthly fever cases (OR: 1.31 [*P* < 0.001], 95% CI: 1.29–1.34; for each 1 standard deviation increase in fever cases), national T&T policy (OR: 336.83 [*P* < 0.001], 95% CI: 257.47–348.01), and pilot phases (OR: 257.22 [*P* < 0.001], 95% CI: 194.29–348.01) were all associated with increased ACT usage (Table 3). There was also a time effect: accumulating experience with positive tests (RDTs and microscopy) increased the probability of using ACTs over other antimalarials (OR: 1.33 [*P* < 0.001], 95% CI: 1.32–1.35). We emphasize that only treating fever cases that test positive for malaria is not mutually exclusive with complete usage of ACTs. Non-ACTs were still administered after health providers fully relied on parasitological diagnosis, suggesting that ACT consumption is not an accurate metric of T&T uptake.

## Discussion

Our modeling results showed that adoption of malaria T&T policies can occur swiftly and completely once initial health provider reluctance is overcome. Experience with positive RDT results was pinpointed as a significant predictor in adherence to test results, which was followed by the abandonment of presumptive diagnosis. The monitoring methods that we developed focus on key decisions of health providers and allowed accurate evaluation of behavior change following T&T introduction (Figure 2). Evaluating these decisions and factors that influence them, rather than ACT or RDT consumption, better quantifies T&T uptake and highlights key factors contributing to adoption of T&T.

Other studies across a diversity of epidemiological and social settings have emphasized inefficiencies in T&T adoption.^10^,^29^ Our study demonstrates successful uptake of the T&T strategies, and importantly, show that a delay in initial uptake of testing does not indicate that complete adoption of T&T behaviors will not occur, confirming another study showing improvement years after T&T introduction.^30^ Many T&T evaluation studies have been conducted over a relatively short period (12–18 months), which is most likely inadequate to demonstrate T&T uptake.^12^,^31^ If other areas follow similar behavioral transitions as Senegal, then change is characterized by a rapid switch in behavior after an initial delay that may last years (Figure 3). Long-term monitoring is essential to determine the success of T&T, and more research should focus on the inertia contributing to initial delays.

With T&T guidelines, practitioners must first decide whether to test a patient for malaria and then, presumably on the basis of the test result, decide whether and with what to treat the patient. Measuring health providers propensity to test a febrile patient (*P*(*test*∣*fever*)) was not a good indicator of T&T adoption because testing of febrile cases was complicated by the realization that many fevers are not caused by malaria. In the clinics we evaluated, adherence to test results (*P*(*treat*∣*neg. test*) = 0) was the first indicator that health providers are adopting T&T policies. This is a better indicator of T&T adoption than ACT or RDT consumption and is fortunately relatively simple to monitor. Reliance on tests for malaria diagnosis (*P*(*treat*∣*no test*) = 0), or abandonment of presumptive diagnosis, followed after full adherence to RDT results was achieved.

By looking at several types of provider responses to T&T policies, we find that policy makers, as well as implementing and funding agencies, should focus on adherence and reliance on parasitological tests rather than ACT or RDT consumption to monitor the successful implementation of T&T. We clearly showed that adoption of T&T clinical behaviors before ACT usage was 100% (it is actually never achieved), and also demonstrated that treatment of untested and negative results with ACTs inflates this metric. In addition, usage statistics of RDTs is problematic because adherence to these results varies greatly.^8^–^11^ RDT consumption reflects the act of testing, rather than adherence to the result.

Furthermore, these modeling results revealed factors that are important in influencing crucial behavioral changes. Consistent across all behaviors, increased exposure to positive parasitological test results allow health providers to reevaluate their perceived risk and adopt T&T strategies. This increased experience (measured as accumulation of positive RDTs and/or microscopy results) was a better predictor than simply time since policy introductions or beginning of pilot studies. Accumulation of positive RDTs, which measures exposure of practitioners to positive results, significantly improved both adherence to test results and reliance on parasitological diagnosis. Exposure to microscopy results increased testing of febrile cases, but did not have a direct effect on the crucial behaviors of adherence or reliance. Resources should be spent encouraging adherence to test results by exposing health practitioners to tests results through workshops and incentives for using RDTs within their clinic.

Several policies were influential in affecting testing and treatment behavior, although not always in their intended manner. National policies increased testing rates, most likely driven by an increase in testing in non-pilot districts. Pilot phases, which were a precursor to national guidelines, were effective in encouraging testing and treating individuals, but the result of the test (or the absence of a test) did not influence the decision to treat. Thus, the pilot phases were not necessary for T&T adoption—in our study they encouraged overtreatment. Pilot phases were not monitored for adherence, which may influence their effectiveness, and more operational research is needed before any particular claims are made about the effectiveness of pilot phases. Finally, it is of importance that freely available RDTs and ACTs were not significant in the adoption of T&T policy. T&T policies were already adopted in most of the districts when resources were made freely available and were not significant in the adoption of these policies.

Although external factors affected T&T adoption (i.e., national T&T Policy, RDT availability), local factors were still very important in influencing all decisions. Seasonal factors, including anomalies in monthly rainfall and fever cases, influenced health decisions. Testing decisions follow rainfall, which varies little between years but has very distinct seasonal patterns within a year (Supplemental Figure 1), but treatment decisions follow monthly fever cases, which exhibit similar seasonality but a decreasing trend over time in some districts (Supplemental Figure 2). Both factors are noninvasive assessments of malaria risk and without parasitological confirmation (or doubt in tests results); health providers can use these metrics as indicators of malaria risk. These seasonal factors had a significant influence on decision making, especially in the absence of parasitological diagnostics, and may contribute to the long lag time in adoption of T&T.

An unfortunate unintended consequence of adoption of T&T policies is the over prescription of antibiotics.^3^,^29^ Although the majority of negative malaria tests are no longer treated with antimalarials beginning in 2006 (Figure 5), there is a significant increase in antibiotics (amoxicillin, doxycycline, ampicillin, cloxacillin, tetracycline, and others). This increase is unsurprising given global trends in antibiotic consumption,^32^ but is a cause for concern. Blanket prescriptions for antibiotics should be avoided to preserve antibiotic efficacy and prolong development of local resistance. We do not know the true causative agents of fevers in this region and more work should be carried out to determine appropriate diagnosis and treatments in lieu of declining malaria.

This study is not without limitations. We are unable to test if stock outs play an important role in T&T adoption, as has been reported in other studies,^33^,^34^ and is most likely a significant challenge for many areas where T&T is being championed. Our data also does not allow us to determine the effect that the policy has had on overall morbidity for malaria. Although malaria incidence dropped dramatically over time and more so concomitantly with the use of ACT,^13^ we cannot be certain that there is a causal relationship between adoption of T&T and decreased malaria incidence. If malaria continues to decline, and testing rates are of only a portion of febrile patients, increasingly rare malaria cases may be missed despite the high sensitivity and specificity of the histidine-rich protein 2 (HRP2)–type RDTs (used in Senegal).^35^ Missed malaria cases pose a challenge for malaria eradication and may eventually affect confidence in T&T policies and lead to practitioners reverting to presumptive diagnosis.

Our analysis relied on data from a small number of clinics, despite this, we anticipate the results to hold for many other clinics. The difference between the districts was no greater than the variability within each district (Table 2). Models suggest consistent and significant predictors, even though there is heterogeneity in individual and clinic-level responses to new policies and individual patient symptoms affect eventual diagnosis and treatment.^36^ Complete T&T adoption occurred in Senegal with an increase in experience with positive malaria tests, along with national policies and RDT availability.

National T&T policies are widespread among malaria endemic countries, but adherence to these policies is limited. Our results are the first to demonstrate full adoption of T&T policies for malaria by individual health clinics. Although there may be delays from RDT availability to initial usage, our model results suggested that belief in the accuracy of RDTs and treatment based on RDT results was a critical factor in the effectiveness of T&T policies and lead to quick abandonment of presumptive treatment. Experience with positive RDT results was a significant predictor of adoption of T&T policies; therefore, focusing efforts on promoting adherence to parasitological tests through practice with RDTs will accelerate T&T uptake and reduce overdiagnosis.

Delays in consistent testing and poor adherence following RDT availability should prompt interventions targeted at behavior of individual providers. Policymakers, malaria program managers, and implementing and funding agencies should focus on this lag between RDT availability, initial provider uptake, and the rate of adherence to RDT results when they are used. Further research is necessary to understand the social and behavioral factors driving delays from RDT availability to initial uptake. Such research will be critical for designing education and incentives that can effectively encourage early RDT usage and adherence. This focus should limit overtreatment, improve T&T adoption, and reduce costs as countries move toward the new Test, Treat, Track directives of WHO.

## Supplementary Material

Supplemental Information.

## Figures and Tables

**Table 1:** Summary of dispensaries

Dispensary	Population	Data availability	Introduction of RDTs	Introduction of ACTs	Health-seeking behavior (consult/person/year)	Wet season fevers, earliest* (fevers/person/month)	Wet season fevers 2011 (fevers/person/month)
Mlomp	7,600	Jan-00	Jan-07	Jan-00	0.73 (0.67–0.79)	0.52	0.14
Djembereng	2,858	Jan-00	Jan-07	Oct-02	0.86 (0.79–0.92)	0.29	0.15
Elinkinde	3,547	Sept-01	Oct-07	Jan-06	0.86 (0.78–0.93)	0.41	0.12
Kabrousse	3,489	Jan-06	Jan-07	Jan-06	1.17 (1.11–1.24)	0.36	0.13
Oussouye†	7,883	Aug-05	Jan-07	Aug-05	0.33 (0.28–0.36)	0.10	0.07
Toucar	3,150	Dec-01	Nov-07	May-06	1.42 (1.26–1.57)	0.47	0.76

ACTs = artemisinin combination therapy; RDTs = rapid diagnosis tests.

This table summarizes the data available from each dispensary. All health clinics are in Oussouye district, except Toucar, which is in Niakhar district.

* Wet season fevers were calculated as the average number of fevers during the earliest wet season with complete data.

† Oussouye has three dispensaries, we only used the Tri dispensary as it had the most complete dataset. This is why the consultation load is so much smaller than the other dispensaries.

**Table 2:** Best-fit multivariate models for monthly clinical behaviors observed. The covariates of the multivariate GLM with the lowest AIC score are listed

Variable	*P*(*test*∣*fever*)	*P*(*treat*∣*neg. test*)	*P*(*treat*∣*no test*)
	OR (95% CI)	OR (95% CI)	OR (95% CI)
Intercept	0.005** (0.004–0.006)	0.05** (0.03–0.07)	34.07** (29.16–39.98)
Epidemiological			
Fevers (anomalies)†	−	1.90* (1.75–2.07)	1.19** (1.16–1.22)
Environmental			
Rainfall (anomalies)†	1.13** (1.11–1.14)	−	−
Policy			
Pilot phase (binary)	73.16** (58.66–92.64)	28.00** (22.45–35.09)	1.30** (1.15–1.46)
T&T policy (binary)	16.72** (13.39–21.18)	−	−
Testing			
RDT availability (binary)	5.60** (5.05–6.22)	4.19** (3.22–5.46)	0.47** (0.41–0.54)
Accum of pos. RDTs‡		0.55** (0.53–0.58)	0.60** (0.58–0.62)
Accum of pos. tests‡	1.17** (1.16–1.18)	−	−
Binary RDT Accum pos. tests*	0.80** (0.79–0.81)	−	−
Dispensaries			
Djembereng	−	−	−
Elinkinde	0.39** (0.35–0.43)	1.05 (0.60–1.82)	1.02 (0.85–1.24)
Kabrousse	1.38** (1.28–1.48)	1.38 (0.60–2.86)	3.10** (2.52–3.83)
Mlomp	2.39** (2.26–2.52)	30.31** (21.07–43.57)	0.031** (0.027–0.036)
Oussouye	1.01 (0.92–1.12)	47.03** (30.94–71.57)	0.38* (0.30–0.47)
Toucar	0.98** (0.91–1.05)	0.21** (0.07–0.50)	3.10* (2.43–3.95)

Accum of pos. = accumulation of positive; AIC = Akaike information criterion; CI = confidence interval; GLM = generalized linear model; neg. = negative; OR = odds ratio; RDTs = rapid diagnosis test; T&T = test and treat policy.

Significance is indicated at *P ≤ 0.05; **P ≤ 0.001.

† Anomalies are determined by the standard deviations around the mean (see Supplemental Information and Supplemental Figures 1 and 2).

‡ Accumulation is determined by dividing the number of tests in the clinic at that date divided by all tests given in the study period and multiplied by 10. One unit represents 10% of the total number of tests. Used instead of raw numbers to normalize across clinics (Supplemental Figure 3).

**Table 3:** Best-fit model for probability of ACT administered as antimalarial

Fixed effects	*P*(*ACT*∣*antimalarials*)
	OR (95% CI)
Intercept	0.0005** (0.0003–0.0006)
Epidemiology	
Fever (anomalies)	1.31** (1.29–1.34)
Policy	
T&T policy (binary)	336.83** (257.47– 348.01)
Pilot phase (binary)	257.22** (194.29–348.01)
Testing	
Accum pos. tests	1.33** (1.32–1.35)
Dispensaries	
Djembereng	
Elinkinde	16.12** (13.98–18.61)
Kabrousse	7.71** (6.82–8.73)
Mlomp	0.25** (0.23–0.28)
Oussouye	15.10** (12.40–18.48)
Toucar	3.27** (2.90–3.70)

Accum of pos. = accumulation of positive; ACT = artemisinin combination therapy; CI = confidence interval; OR = odds ratio; T&T = test and treat policy.

The covariates of the multivariate generalized linear model (GLM) with the lowest Akaike information criterion (AIC) score were fit for *P* (ACT/antimalarials).

Significance is indicated at **P ≤ 0.001.
