# Supplementary material for: Assessing Drivers of Full Adoption of Test and Treat policy for Malaria in Senegal
Source: Am J Trop Med Hyg. 2015 Jul 8;93(1):159–67. doi: 10.4269/ajtmh.14-0595 (PMC4497889; doi:10.4269/ajtmh.14-0595)
Supplement: Supplementary file 1 [file SD6.pdf]

SUPPLEMENTAL INFORMATION

METHODOLOGY

Fitting a logistic curve to transitions. A logistic model was used to describe the change from a non–rapid diagnosis test (-RDT) regimen to full-RDT implementation for two of the conditional relationships: $P(treat|no\ test)$ and $P(treat|neg.\ test)$. The other conditional relationships dealing with testing behavior ($P(test|fever)$) and treatment decisions ($P(ACT|antimalarials)$) did not saturate, and thus a logistic model was not fit to these data.

In this model, we denoted t to be the time in months since the introduction of the policy, A to be the growth rate, and B to be the midpoint of the transition. Parameters A and B were estimated via Markov chain Monte Carlo (MCMC) using PyMC 2.3[1] in Python 2.7.4.[2]

$$f(t)=\frac{1}{1+e^{-A(t-B)}} \tag{1}$$

Because the timing of RDT introduction and ACT availability varied between dispensaries, logistic models were fit to each unique dispensary. Parameters were estimated for $P(treat|no\ test)$ and $P(treat|neg.\ test)$.

Data for two clinics were available for every month from 2000 to 2011 (Mlomp and Djembereng), while the remaining clinics covered subsets of this period (Table 1).

Logistic regression model selection. Logistic regression models were created using the generalized linear model function (*glm*) in R 2.17.[3] This function fits models via maximum likelihood; therefore, Akaike information criterion (AIC) goodness-of-fit measure was used for model comparison and selection.[4] Best-fit models were chosen for each conditional relationship tested: 1) $P(test|fever)$, 2) $P(treat|no\ test)$, 3) $P(treat|neg.\ test)$, and 4) $P(ACT|antimalarials)$. A note about $P(ACT|antimalarials)$: the recommended treatment in the region is ASAQ, but other ACTs are available as well (artemether/lumefantrine, artesunate/mefloquine, and dihydroartemisinin/piperaquine). We combined all ACTs into a single binary variable; non-ASAQ ACTs made up a small percentage of the total ACTs prescribed (2.95%, 426/14,439).

We considered statistical differences to be significant if P values were lower than 0.05.

Covariates used in logistic models. *Environmental.* For the district, we had monthly rainfall (mm) data. In the dry season (November–May), no rainfall occurred except for November 2009 (2 mm), whereas the rainfall in the rainy months (June–October) ranged from 21 to 759 mm. Rainfall was then transformed into standard deviations from the mean (109.55 mm). Thus, monthly rainfall was interpreted as an anomaly from the average monthly rainfall.

Epidemiological. From each clinic, we had access to number of patients that came in each month for consultations. Consultations were used as a proxy for how busy the clinic was in a given month, although we acknowledge that the cases were not evenly dispersed over each day. These data were included as raw numbers and as quartiles calculated for each dispensary. Each dispensary only had one health provider, so even though their case loads were different across the clinics, we believe that each health provider was using his local cues (i.e., number of consultations that month at his/her clinic) rather than global cues (i.e., average consultations across all clinics). Among the consultations, subsets were febrile, and these numbers were used as monthly fever cases; both raw numbers and anomalies for each district were used (Supplemental Figure 2).

Policy. National T&T policy was enacted in January 2007. This policy was incorporated as a binary variable (0 for pre-2006, and 1 for any time after the policy) and as time since the policy was enacted (starting with 1 at January 2006 and going up to 71 months in December 2011). This was the only covariate that is consistent in across all clinics, as timing of other events (i.e., RDT availability) and caseloads (i.e., fevers) varied between months and clinics. Pilot phases for T&T of malaria cases took place in Djembereng from 2002 to 2005 and Mlomp from 2000 to 2005. Pilot phases introduced parasitological diagnosis (by microscopy) and the new treatment (ASAQ) available to clinics. Health practitioners were instructed to test suspected malaria cases and give ASAQ to patients with a positive test. This covariate was included in the model as binary— indicating an active pilot phase or inactive. Districts that did not have a pilot phase were "0" through the whole period.

In addition to a switch from presumptive diagnosis to a policy of T&T, the recommended treatment regimen changed over the course of this time series (Figure 1). Each treatment

SUPPLEMENTAL TABLE 1
Accumulation of positive parasitological tests included in the models

Districts	RDTs	Tests (RDTs plus microscopy)
Djembereng	94	188
Elinkinde	23	23
Kabrousse	142	142
Mlomp	68	684
Oussouye	20	20
Toucar	72	72

RDTs = rapid diagnosis tests.
Testing rates varied greatly between districts and over time. To quantify a comparable metric for exposure to test results, we converted number of tests and number of positive tests to a district-specific accumulation rates. The cumulative number of tests each month was divided by the total number of tests for that district over the entire study period. This was then multiplied by 10 to make the model results more interpretable. In all models, accumulation of positive parasitological test results (either rapid diagnostic tests [RDTs] or RDTs plus microscopy) were better predictors than the adjusted number of tests (positive and negative test results). The table includes the rounded number of individual positive tests that translate to a 10% accumulation in each district. Note that in non-pilot district the accumulation of RDTs and tests are equivalent.

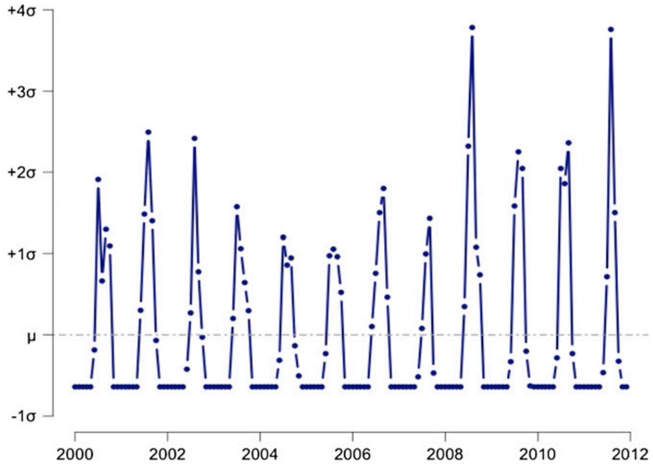

SUPPLEMENTAL FIGURE 1. Rainfall anomalies over the study period. Monthly rainfall data were collected and used to determine anomalies over the study period (μ = 109.55 mm, σ = 170.88 mm).

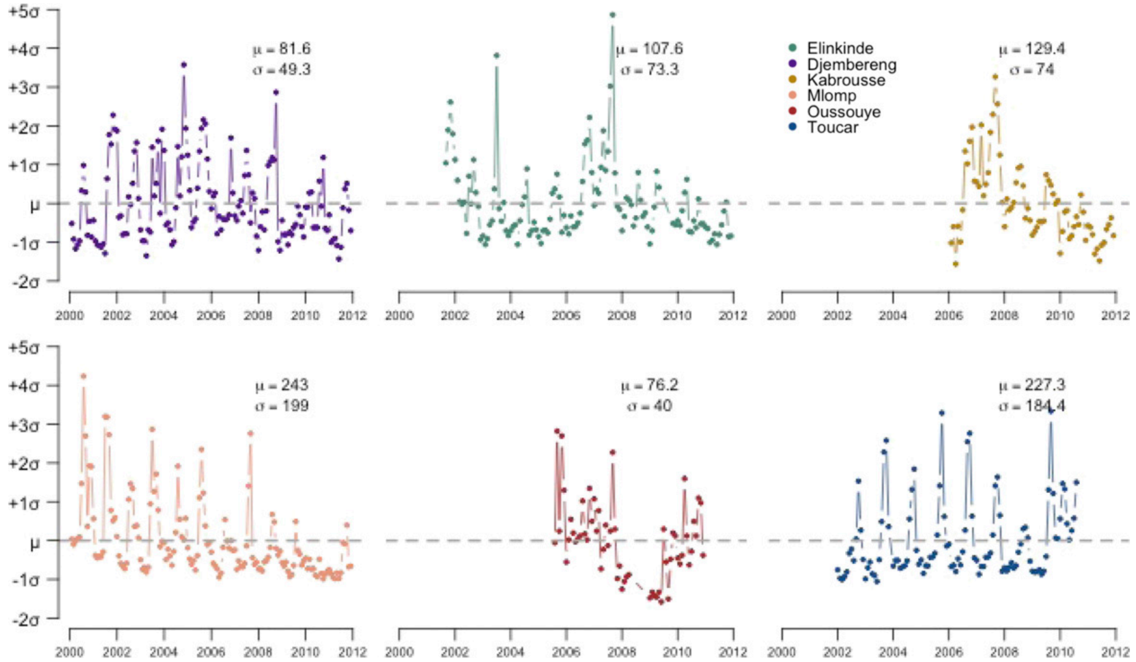

SUPPLEMENTAL FIGURE 2. Monthly fever anomalies. The monthly number of fever cases at a dispensary was normalized across the districts by calculating the number of standard deviations from the total mean for each dispensary. The average and spread of monthly fever cases varied between and among dispensaries and showed different trends over time.

regimen was designated as a separate binary covariate. For times when a particular drug was the recommended first-line treatment, the value was "1." ASAQ and T&T policy completely overlapped, so it was included only as a binary variable of the national T&T policy. However, free ASAQ was included as a binary covariate in some models.

Testing. Clinics had access to parasitological diagnosis by microscopy, but turnaround time and expense prevented health practitioners from using microscopy. However, during pilot phases, many practitioners did use microscopy, and training emphasized that pilot phase health practitioners should prioritize children when ordering microscopy. RDTs

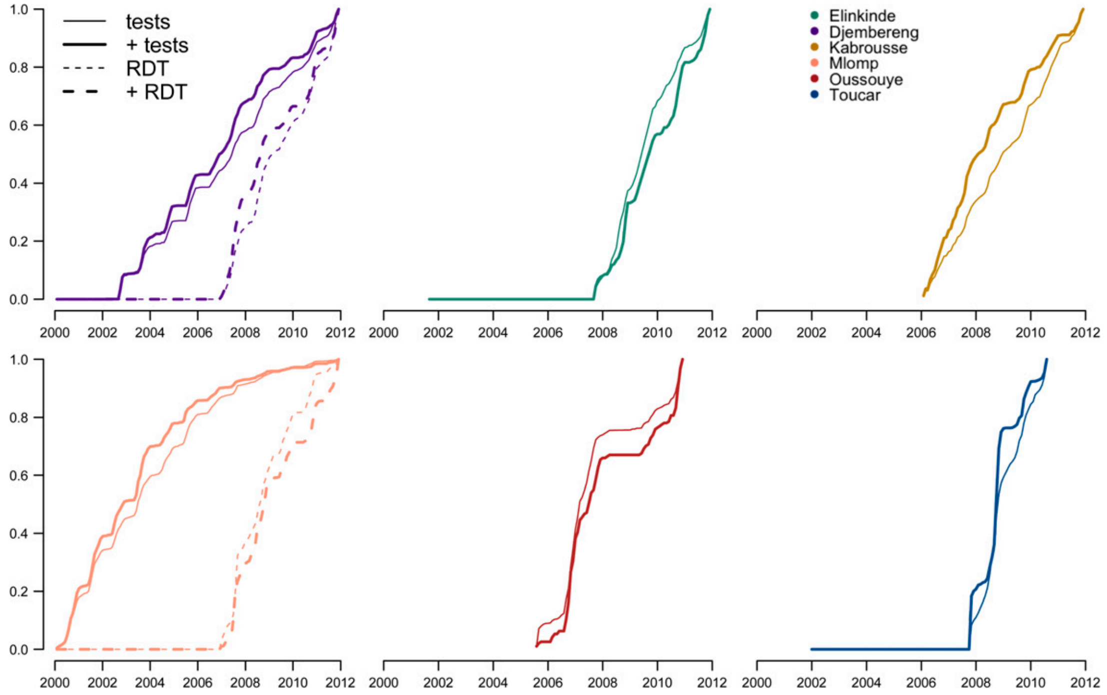

SUPPLEMENTAL FIGURE 3. Accumulation of test results. Each figure represents a unique dispensary and shows the accumulation of test results, either microscopy plus RDT (solid) or RDT alone (dashed), over the study period. Some dispensaries only had a set of lines because microscopy was not used for testing prior to the introduction of RDTs.

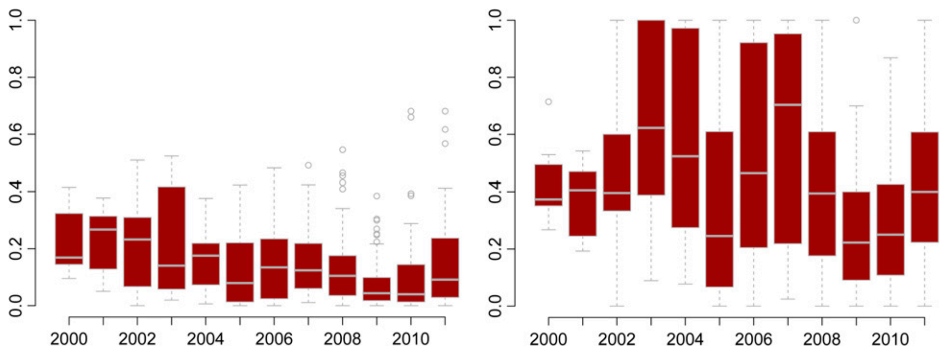

SUPPLEMENTAL FIGURE 4. Proportion of positive tests. (**A**) The proportion of febrile cases that have a positive malaria test decreases overtime, although the variance is quite high. This data reflect all districts and variability in testing behaviors in addition to variability in malaria incidence. (**B**) The proportion of positive cases from tested febrile cases is much more variable—this is most likely correlated with increased testing from 2003 to 2007, and reflects both wet and dry season test results.

(histidine-rich protein 2 [HRP2]) were made available to dispensaries beginning in 2007, but the actual date of delivery varied between January and October 2007. We assumed that clinics had full access to RDTs and no stock outs occurred once they were made available. These data were included as covariates in the model—either as binary or time (in months) since RDTs were available. Once testing became available, we also used the accumulation of positive test results as a model covariate. We used this variable because although RDTs might be available in a district, usage may not be linear overtime. Instead, as trust in the test accumulates, the use may increase exponentially. Accumulation of test results was normalized so that each month was a proportion of the total tests in that district (Supplemental Figure 3).

In addition to RDT availability, we also kept track of freely available tests. In 2010, both RDTs and ASAQ treatments were freely available for all patients. We incorporated this into models as a binary variable: "1" represented periods when RDTs were free and "0" were times before 2010 when RDTs cost.

REFERENCES

1. Patil A, Huard D, Fonnesbeck C, 2010. PyMC 2.0: Bayesian stochastic modelling in Python. *J Stat Softw 35:* 1–81.
2. Python Software Foundation, 2010. *Python Language Reference, Version 2.7.* Available at: http://www.python.org.
3. R Core Team, 2013. *R: A Language and Environment for Statistical Computing*. Vienna, Austria: R Foundation for Statistical Computing. Available at: http://www.R-project.org/.
4. Burnham K, Anderson D, 1998. *Model Selection and Inference: A Practical Information–Theoretic Approach*. New York, NY: Springer-Verlag.
